# A New Appliance for Class III Treatment in Growing Patients: Pushing Splints 3

**DOI:** 10.1155/2019/9597024

**Published:** 2019-11-11

**Authors:** Stefano Martina, Roberto Martina, Lorenzo Franchi, Vincenzo D'Antò, Rosa Valletta

**Affiliations:** ^1^Department of Medicine, Surgery and Dentistry, “Scuola Medica Salernitana”, University of Salerno, Via Allende, 84081 Baronissi, Italy; ^2^School of Orthodontics, Department of Neurosciences, Reproductive Sciences and Oral Sciences, University of Naples Federico II, Via Pansini 5, 80131 Naples, Italy; ^3^Department of Experimental and Clinical Medicine, University of Florence, Largo Brambilla 3, 50134 Florence, Italy

## Abstract

Several orthopedic procedures have been used in early treatment to reduce the need for orthognathic surgery in skeletal Class III. The most used treatment is Rapid Maxillary Expansion and Facemask. This procedure also determines a clockwise rotation of the mandible, increasing the vertical dimensions of the lower third of the face. Therefore, the control of vertical dimension appears to be a key objective in Class III hyperdivergent patients. This article shows two skeletal Class III patients treated with a new appliance (Pushing Splints 3), that is able to correct sagittal discrepancy with a good control of the vertical growth. In both cases, Class I relationship with a proper Overjet and Overbite was achieved with improvement of profile. The final cephalometric values demonstrated a stable sagittal relationship and a good control of the vertical growth. The specific biomechanic features of the PS3 appliance permit the improvement of the sagittal jaw relationship, delivering at the same time vertical vectors that are able to control the alveolar and skeletal components of the vertical growth. This could be useful in the treatment of Class III hyperdivergent patients.

## 1. Introduction

Skeletal Class III is one of the most challenging malocclusions for the orthodontist. The skeletal and dental components of Class III malocclusions are usually present since early childhood [1], and an early treatment is usually suggested to avoid or to reduce the need for an orthognathic surgery [2].

Several orthopedic procedures have been used to reach this purpose in early treatment such as Fränkel III [3], chin cup [4], mandibular reverse headgear, and bone-anchored maxillary protraction [5].

Currently, the most frequently used treatment procedure involves the combination of Rapid Maxillary Expansion and Facemask [6, 7], but there is still a need of high-quality evidence about the effectiveness of this treatment, particularly regarding long-term stability [8]. On the contrary, many authors demonstrated that the desired forward movement of the maxilla is accompanied by a downward mandibular movement which also determines a clockwise rotation of the mandible. The overall effect appears to be an increase in vertical dimensions of the lower third of the face that is obviously inappropriate for patients with increased vertical skeletal relationships [8].

Therefore, the control of vertical dimension appears to be a key objective in Class III hyperdivergent patients.

This article describes the use of a modified version of the SEC appliance originally presented by Ferro et al. [9].

## 2. Appliance Design

The appliance (Pushing Splints 3, PS3) consists of three components: two acrylic splints and a Forsus™ L-pin module per side.

The two splints are built-up through a traditional acrylic appliance construction procedure by a dental technician. A 2 mm high construction bite is used in order to leave the space necessary to have a flat occlusal plane on both of the splints.

**Figure 1:**
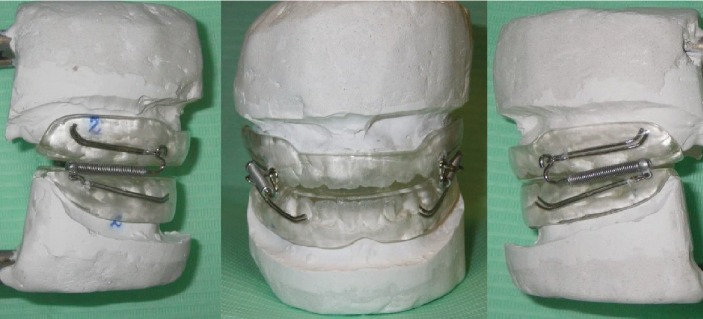
PS3 appliance.

The two splints cover all the tooth crowns—usually from the left first permanent molar to the right first permanent molar 6 to 6—in both arches. The Forsus™ L-pin modules are used in order to deliver a force of 200 g per side in a forward direction to the upper splint and in a backward direction to the lower splint. In an opposite way from Class III elastics, the vertical component of the force delivered by the Forsus™ L-pin module is directed upward and forward in the maxilla and downward and backward in the mandible (Figure 1). Working with a pushing system is important to obtain a good retention of the splint even if some grinding is needed now and then to avoid interference with the eruption of permanent teeth.

However, the really important advantage of PS3's force system is the control of the vertical growth.

Patients are instructed to use the splints as much as possible, with a minimum time of 14 hours per day, which probably exceeds the average wear time of the Facemask, whose wearing is certainly more invasive than the intraoral splints.

## 3. Case Report No. 1

**Figure 2:**
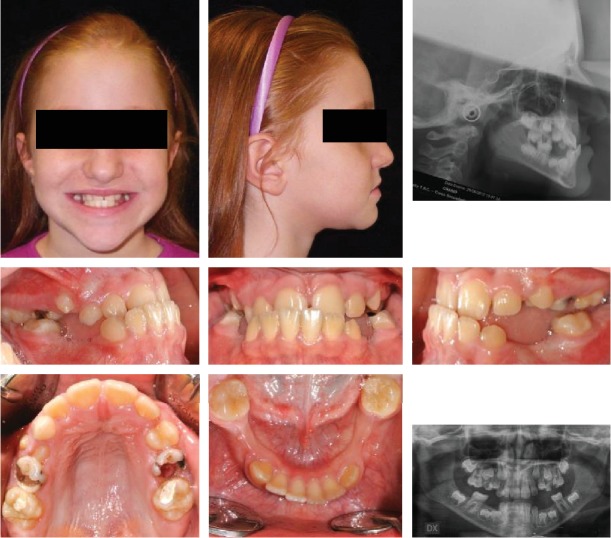
Case no. 1 facial photos, intraoral photos, panoramic radiograph, and lateral cephalogram at *T*_0_ (pretreatment).

The patient was an 8.0-year-old female with a Class III dental and skeletal (A‐N‐Pg = −4.5°) malocclusion. A bilateral posterior crossbite and a midline discrepancy were also present. Due to decay, 7.4, 7.5, 8.4, and 8.5 were extracted (Figure 2).

**Figure 3:**
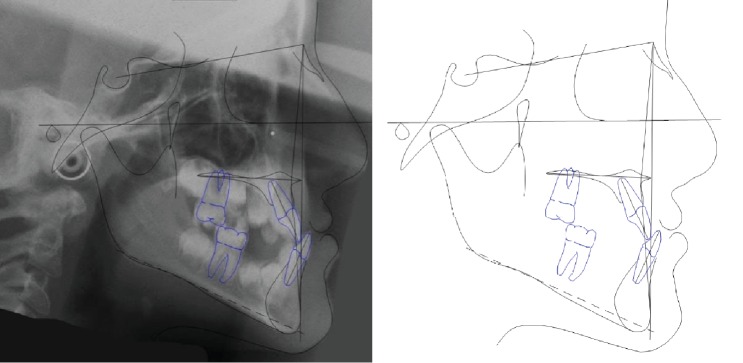
Case no. 1 tracings (pretreatment).

The increased value of the Sn-GoGn angle (33 degrees) showed a tendency to a hyperdivergent vertical skeletal relationship that was also demonstrated by the condyle neck morphology (Figure 3).

The main treatment objective was to correct the Class III growth pattern. Because of the increased vertical growth, an important treatment objective was also to control the hyperdivergency.

In order to control the vertical growth usually increased by this kind of treatment, a PS3 appliance was used in the orthopedic phase.

The treatment time with the PS3 was 13 months. The appliance was reactivated after 6 months adding a split crimp (1.5 mm) to the push rod bilaterally. The upper splint was once relined to increase its stability.

**Figure 4:**
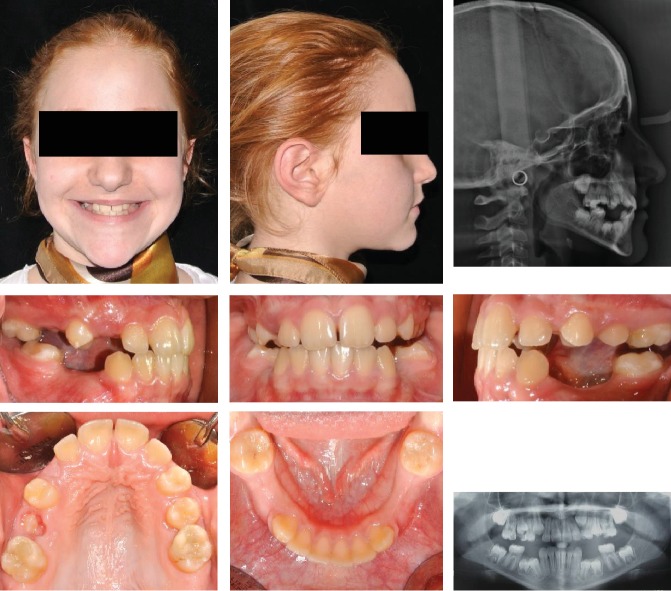
Case no. 1 facial photos, intraoral photos, panoramic radiograph, and lateral cephalogram at *T*_1_ (after PS3 treatment).

After the orthopedic phase of the treatment, the profile greatly improved. Normal values of Overjet and Overbite were also achieved. A tendency to a Class III molar relationship was still present on the right side due to the lower first molar mesial shift because of the early extraction of deciduous molars (Figure 4).

**Figure 5:**
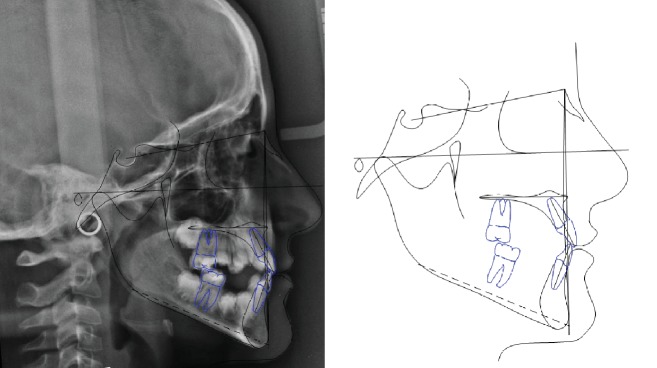
Case no. 1 tracings at *T*_1_.

The sagittal relationship improved (A-N-Pg angle value from -4.5 to -1.7 degrees) and the hyperdivergent growth pattern was under control (Sn-GoGn angle 33.6 degrees) (Figure 5).

**Figure 6:**
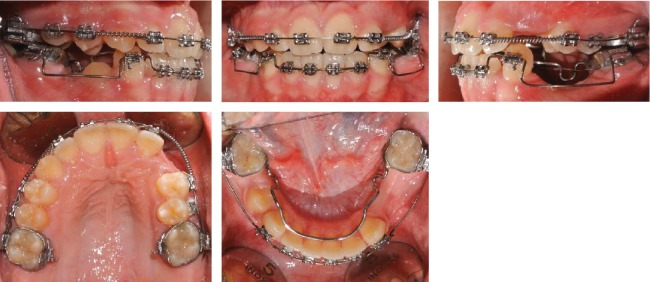
Case no. 1 fixed appliance phase.

Fixed appliances were progressively bonded to both arches with MBT prescription. Bilateral Class III elastics and box elastics with a Class III component were shortly used in the last phase to reach the final occlusion. The fixed appliance phase lasted 24 months as a consequence of the delayed time of 3.4 and 3.5 eruption (Figure 6).

**Figure 7:**
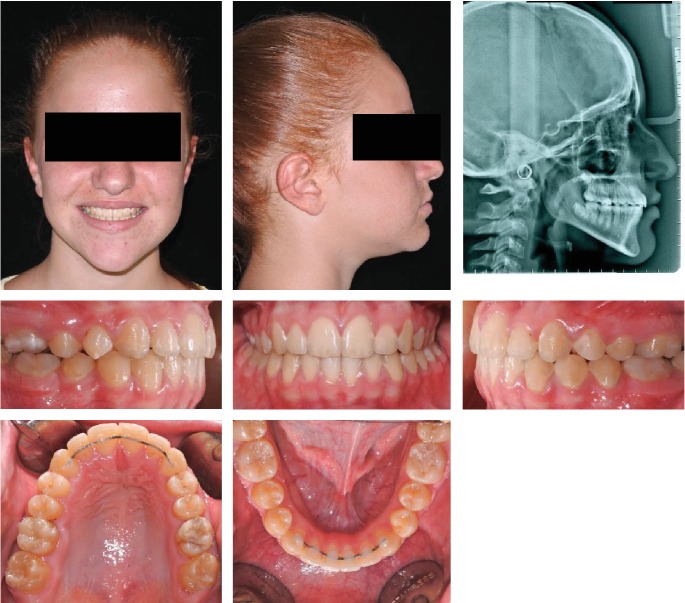
Case no. 1 facial photos, intraoral photos, and lateral cephalogram at *T*_2_ (posttreatment).

A Class I relationship with a proper Overjet and Overbite was achieved. A straight nice profile with a good vertical proportion was also maintained after the pubertal growth spurt (Figure 7).

**Figure 8:**
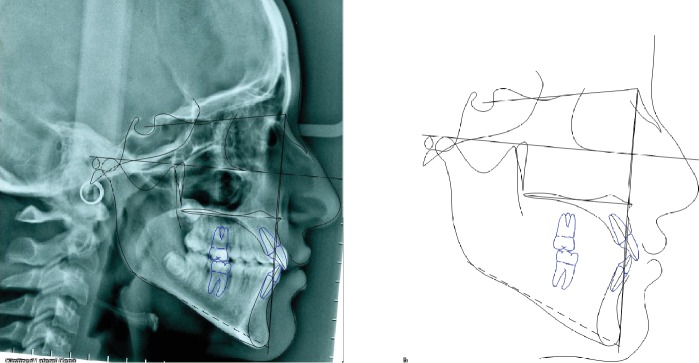
Case no. 1 tracings at *T*_2_.

**Figure 9:**
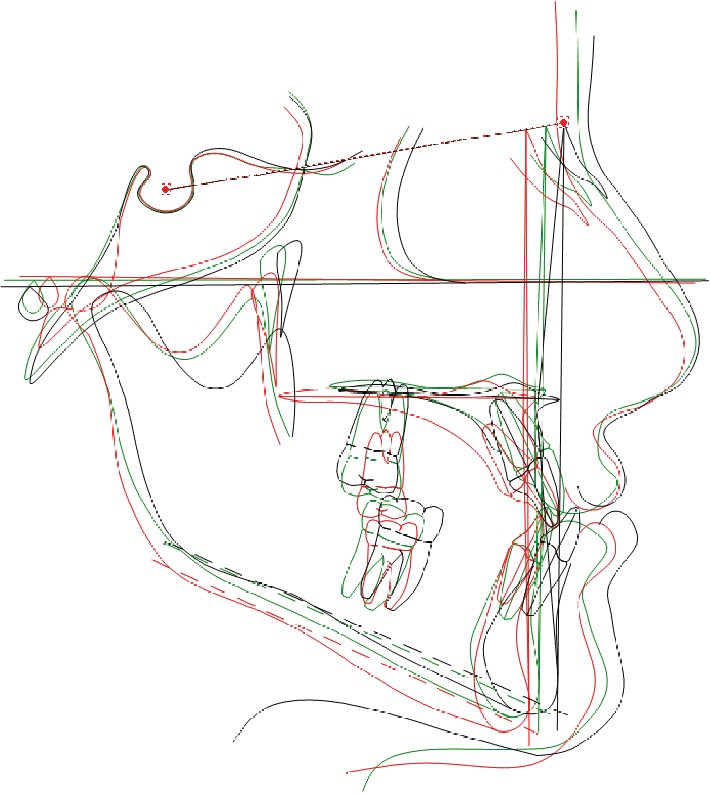
Case no. 1 tracing superimpositions.

The final cephalometric values demonstrated a stable sagittal relationship (ANPg angle -0.8 degree), a good control of the vertical growth also in this phase of treatment (Sn-GoGn angle 34 degrees), and nearly no dental compensation of the lower incisors (Figures 8 and 9) (Table 1).

## 4. Case Report No. 2

**Figure 10:**
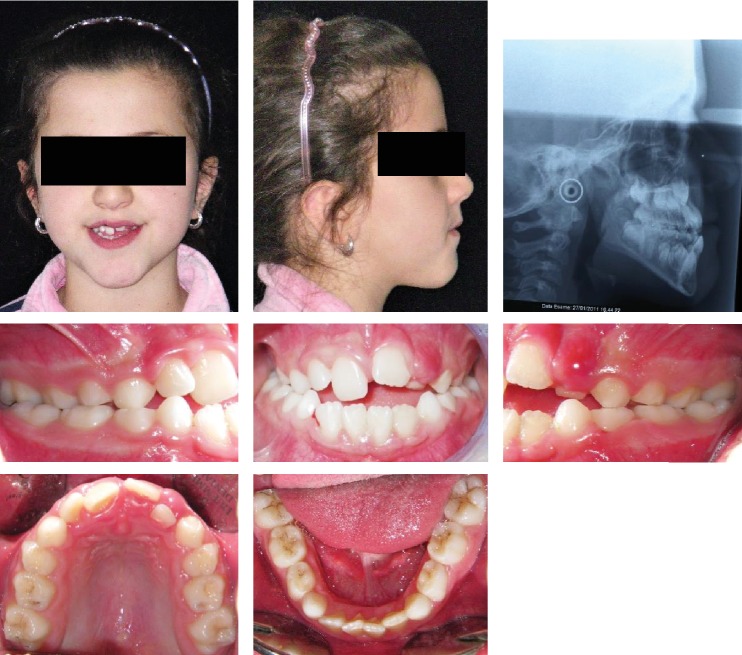
Case no. 2 facial photos, intraoral photos, panoramic radiograph, and lateral cephalogram at *T*_0_ (pretreatment).

The patient was an 8.9-year-old female with a Class III dental and skeletal (A‐N‐Pg = −1.0 degree) malocclusion. A negative Overjet (-1.4 mm) and Overbite (-3.7 mm) were present. A bilateral posterior crossbite was also present (Figure 10).

**Figure 11:**
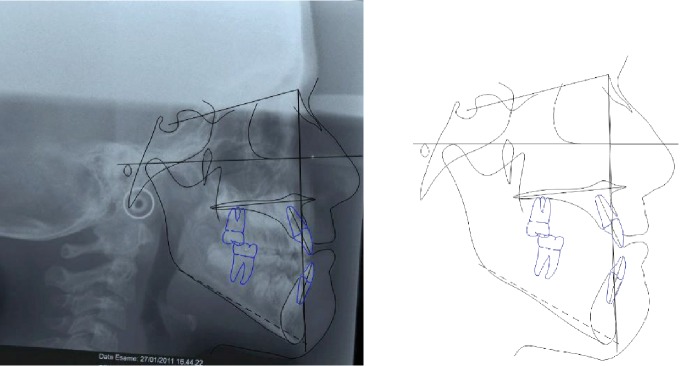
Case no. 2 tracings (pretreatment).

The increased value of the Sn-GoGn angle (43.7 degrees) showed a severe vertical growth pattern. The mandible morphology confirmed a severe structural hyperdivergency (Figure 11).

The profile also corroborates both the skeletal component of the Class III malocclusion and the increased vertical skeletal relationship.

As in the previous case, the treatment objective was to improve the skeletal Class III relationship and to control the hyperdivergency.

An interceptive orthopedic treatment plan with PS3 was proposed with the aim of improving the sagittal skeletal relationship and to control at the same time the vertical dimension.

The treatment time with the PS3 was 16 months. The appliance was reactivated after 6 and 12 months adding a split crimp (1.5 mm) to the push rod bilaterally. The upper splint was once relined to increase its stability.

**Figure 12:**
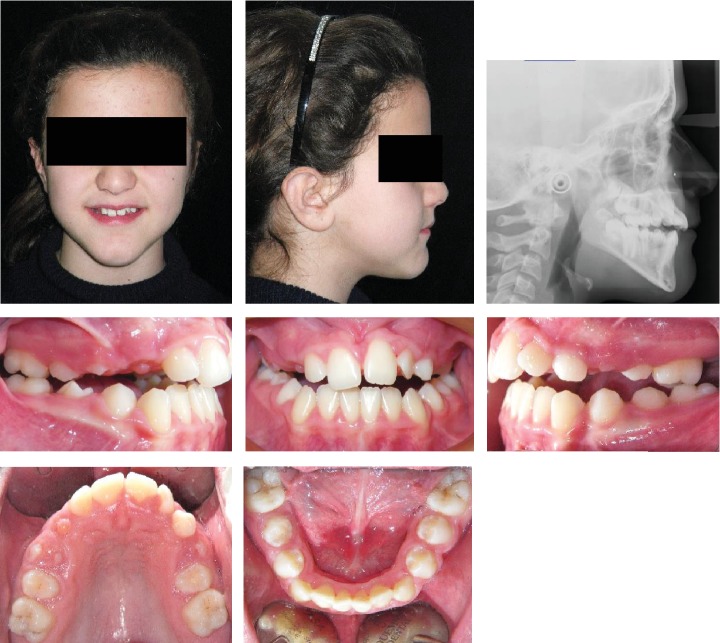
Case no. 2 facial photos, intraoral photos, panoramic radiograph, and lateral cephalogram at *T*_1_ (after PS3 treatment).

After the orthopedic phase of treatment, the profile greatly improved. An overcorrection of the Overjet was obtained (6.7 mm) while the open bite was still present (-2.0 mm). A full Class II molar relationship was reached on the left side, while an edge-to-edge relationship was present on the right side (Figure 12).

**Figure 13:**
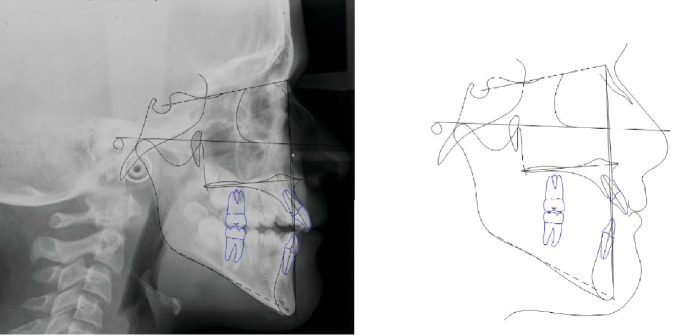
Case no. 2 tracings at *T*_1_.

The sagittal relationship improved (ANPg angle value from -1 to 1.3 degrees). The mandibular inclination decreased (Sn-GoGn from 43.7 to 39.5 degrees) (Figure 13).

A fixed appliance was progressively bonded to both arches with MBT prescription. Bilateral Class III elastics and box elastics with a Class III component were shortly used in the last phase to refine the occlusion. The fixed appliance phase lasted 23 months.

**Figure 14:**
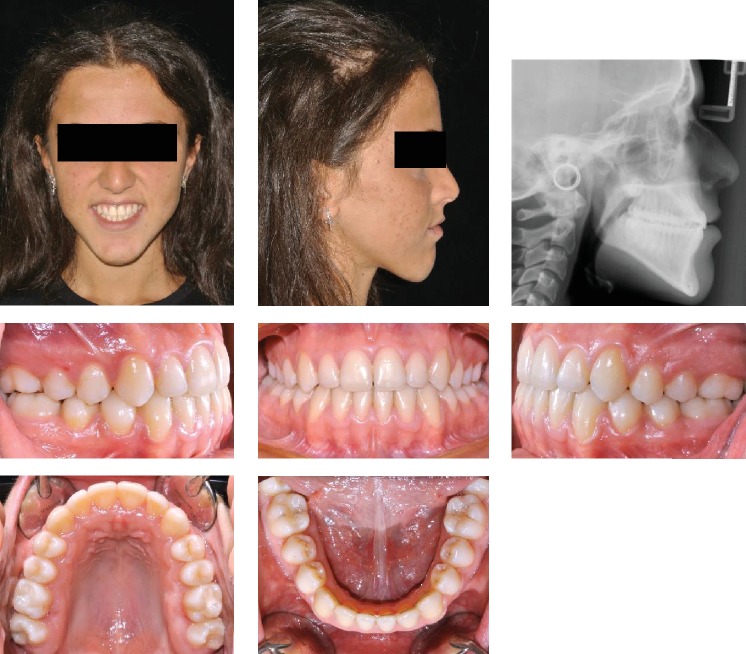
Case no. 2 facial photos, intraoral photos, and lateral cephalogram at *T*_2_ (posttreatment).

A very good Class I intercuspation with a proper Overjet and Overbite eventually was achieved (Figure 14).

**Figure 15:**
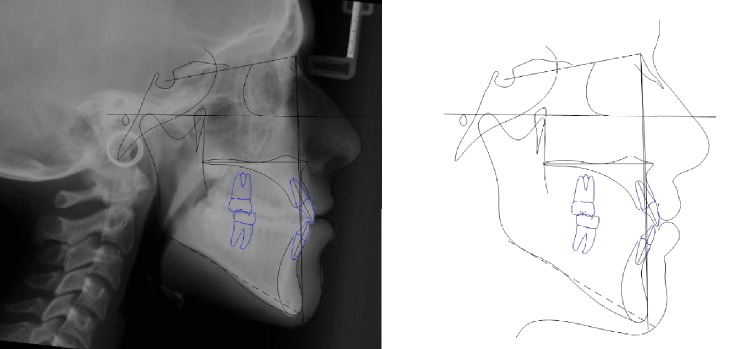
Case no. 2 tracings at *T*_2_.

**Figure 16:**
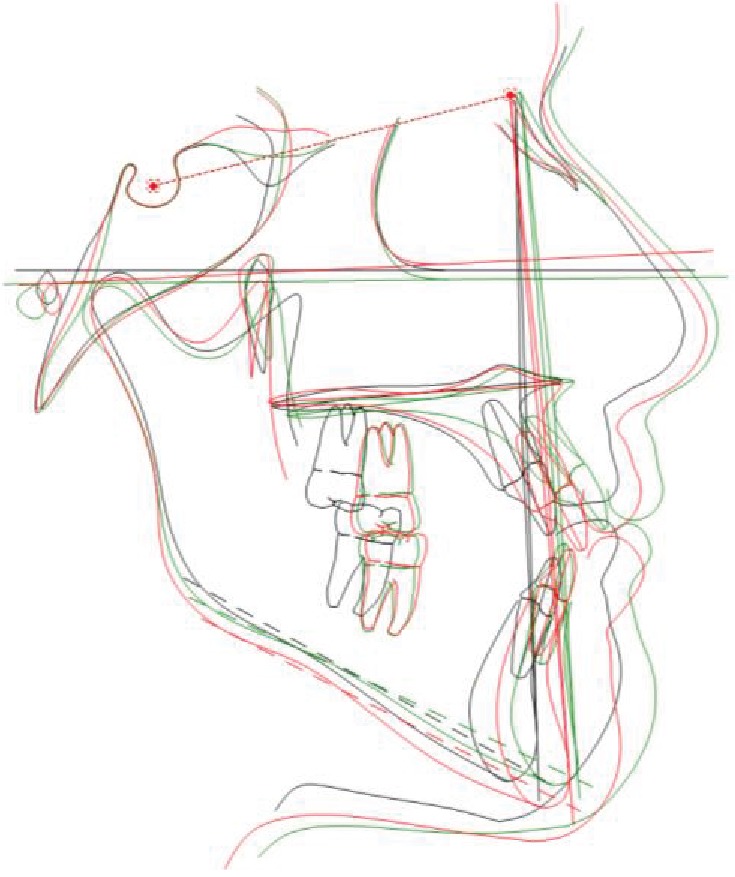
Case no. 2 tracing superimpositions.

The final ANPg angle was 0.9 degree keeping stable a good correction of the sagittal jaw relation. The mandibular inclination increased again to 41.3 degrees (Table 2), but considering the very severe hyperdivergent growth pattern, the final value should be considered as a quite favorable result (Figures 15 and 16).

The profile, however, was affected by the mandibular inclination and morphology. A chin surgery could be considered in case of an esthetic request from the patient.

## 5. Discussion

As widely recognized, Facemask and RPE are still the most utilized treatment procedure for a Class III malocclusion. Some limits should be considered in this protocol: (1) the need for a huge cooperation, (2) space lost in the upper arch due to upper posterior teeth mesial shifting, and (3) the increase in the vertical dimension.

Point (1) is obvious, due to the invasiveness of the use of FM. Furthermore, a more significant orthopedic result is usually obtained with a very early treatment, which generally also corresponds to an even lower level of cooperation. PS3, not needing an extra oral appliance, could facilitate cooperation.

Point (2) is connected to the FM/RPE biomechanics that determines a mesial movement of posterior teeth covered by the acrylic splints. The PS3 splints cover the whole arch, offering a good control of the arch length.

Point (3) is specifically related to the peculiar biomechanics of the FM/RPE protocol whose sagittal activation modality facilitates at the same time a vertical movement of the upper jaw and the upper arch [7]. Rongo et al. [8] showed that FM therapy determined a clockwise rotation of the lower jaw that leads to an increase in vertical skeletal relationships; hence, even if Class III early orthopedic treatment has been demonstrated effective in sagittal correction, it may produce unfavorable side effects in hyperdivergent patients.

The most important advantage of PS3 pushing biomechanics is the good control of upper jaw vertical growth, which is extremely important in hyperdivergent cases, such as those reported in the present article.

Finally, data from a recent paper [10] confirmed that the PS3 protocol is able to improve the sagittal relationship (ANPg *T*_1_ − *T*_0_ = 2.8 degrees, *p* < 0.001) and to preserve clockwise mandibular rotation (Sn-GoGn *T*_1_ − *T*_0_ = 0.6 degrees, *p* > 0.05).

This is a crucial point of difference also with the treatment performed with Class-III elastics in a fixed appliance, since even the elastics biomechanics has the effect of increasing the vertical dimension.

According to Fränkel and Fränkel [3], FR-3 is “an orthopedic exercise device that is capable of overcoming the faulty spatial disorders as well as the faulty postural performances of the orofacial musculature associated with Class III malocclusions” but a long treatment time—at least 5 years—is necessary for the appliance to be effective, which greatly reduces the efficiency of the treatment.

Some other protocols have been suggested in the last years, but they require invasive surgery and some cooperation [5] or some surgery and important cooperation [11]. None of these treatments could anyway guarantee the certainty of avoiding relapse and the need for surgery.

Two systematic reviews [12, 13] reported that Facemask and chin cup did not constitute a risk factor of TMD. Since both these appliances use higher forces than PS3 compressing the TMJ, it could be assumed that PS3 should not cause TMD. This assumption seems to be confirmed by the observation of over a hundred treated cases.

## 6. Conclusions

The control of the vertical dimension appears to be a key objective in Class III hyperdivergent patients.

The specific biomechanic features of the PS3 appliance permit the use of forces useful to improve the sagittal jaw relationship, delivering at the same time vertical vectors that are able to control the alveolar and skeletal components of the vertical growth. This specific biomechanic feature should be considered as a useful benefit in the treatment of severe hyperdivergent patients.

## Figures and Tables

**Table 1:** Cephalometric values of case no. 1 before treatment, after PS3 treatment, and at the end of fixed appliance treatment.

Values	Initial	Progress	Final
SNA (°)	75.2	77.9	79.8
S-N-Pg (°)	79.7	79.6	80.6
A-N-Pg (°)	-4.5	-1.7	-0.8
Co-Gn (mm)	113.6	115.9	117.6
Co-Go (mm)	48.6	51.9	54.9
Co-Go-Me (°)	131.9	136.3	134.3
S-N/ANS-PNS (°)	12.2	10.6	10.1
S-N/Go-Gn (°)	33.0	33.6	34.0
ANS-PNS/Go-Gn (°)	23.3	25.8	26.0
U1-ANS-PNS (°)	117.5	115.9	125.0
L1-Go-Gn (°)	92.1	87.7	90.7
Overjet (mm)	-3.9	2.0	2.7
Overbite (mm)	4.2	0.6	1.7

**Table 2:** Cephalometric values of case no. 2 before treatment, after PS3 treatment, and at the end of fixed appliance treatment.

Values	Initial	Progress	Final
SNA (°)	76.7	81.7	81.6
S-N-Pg (°)	77.6	80.5	80.7
A-N-Pg (°)	-1.0	1.3	0.9
Co-Gn (mm)	114.9	118.7	121.5
Co-Go (mm)	55.4	56.8	62.2
Co-Go-Me (°)	138.2	136.2	136.9
S-N/ANS-PNS (°)	9.0	7.4	10.8
S-N/Go-Gn (°)	43.7	39.5	41.3
ANS-PNS/Go-Gn (°)	37.7	34.3	32.7
U1-ANS-PNS (°)	109.5	118.3	113.9
L1-Go-Gn (°)	77.7	76.4	82.0
Overjet (mm)	-1.4	6.7	2.0
Overbite (mm)	-3.7	-2.0	0.3
